# Minimum Penalized *ϕ*-Divergence Estimation under Model Misspecification

**DOI:** 10.3390/e20050329

**Published:** 2018-04-30

**Authors:** M. Virtudes Alba-Fernández, M. Dolores Jiménez-Gamero, F. Javier Ariza-López

**Affiliations:** 1 Departamento de Estadística e Investigación Operativa, Universidad de Jaén, 23071, Jaén, Spain; 2 Departamento de Estadística e Investigación Operativa, Universidad de Sevilla, 41012, Sevilla, Spain; 3 Departamento de Ingeniería Cartográfica, Geodésica y Fotogrametría, Universidad de Jaén, 23071, Jaén, Spain

**Keywords:** minimum penalized *ϕ*-divergence estimator, consistency, asymptotic normality, goodness-of-fit, bootstrap distribution estimator, thematic quality assessment

## Abstract

This paper focuses on the consequences of assuming a wrong model for multinomial data when using minimum penalized ϕ-divergence, also known as minimum penalized disparity estimators, to estimate the model parameters. These estimators are shown to converge to a well-defined limit. An application of the results obtained shows that a parametric bootstrap consistently estimates the null distribution of a certain class of test statistics for model misspecification detection. An illustrative application to the accuracy assessment of the thematic quality in a global land cover map is included.

## 1. Introduction

In many practical settings, individuals are classified into a finite number of unique nonoverlapping categories, and the experimenter collects the number of observations falling in each of such categories. In statistics, that sort data is called multinomial data. Examples arise in many scientific disciplines: in economics, when dealing with the number of different types of industries observed in a geographical area; in biology, when counting the number of individuals belonging to one of *k* species (see, for example, Pardo [1], pp. 94–95); in sports, when considering the number of injured players in soccer matches (see, for example, Pardo [1], p. 146); and many others.

When dealing with multinomial data, one often finds zero cell frequencies, even for large samples. Although many examples can be given, we will center on the following one, since two related data sets will be analyzed in Section 4. Zero cell frequencies are usually observed when the quality of the geographic information data is assessed, and specifically, when we pay attention to the thematic component of this quality. Roughly speaking, the thematic quality refers to the correctness of the qualitative aspect of an element (pixel, feature, etc.). To give an assessment of the thematic accuracy, a comparison is needed between the label considered as true of a feature and the label assigned to the same feature after a classification (among a number of labels previously stated). This way, each element/feature, which really belongs to a particular category, can be classified as belonging to the same category (correct assignment), or as belonging to another one (incorrect assignment). Given a sample of *n* elements belonging to a particular category, after collecting the number of elements correctly classified, $X_{1}$, and the number of incorrect classifications in a set of k−1 possible categories, $X_{i}$, i=2,…,k, we obtain a multinomial vector ${\left(X_{1},X_{2},\ldots ,X_{k}\right)}^{t}$, for which small or zero cell frequencies are often observed associated with the incorrect classifications, $X_{i}$, i=2,…,k.

Motivated by this example in the geographic information data context, as well as many others, along this paper, it will be assumed that the available information can be summarized by means of a random vector $X={\left(X_{1},\ldots ,X_{k}\right)}^{t}$ having a *k*-cell multinomial distribution with parameters *n* and $\pi ={\left(\pi_{1},\ldots ,\pi_{k}\right)}^{t}\in \Delta_{0k}=\left\{{\left(\pi_{1},\ldots ,\pi_{k}\right)}^{t}:\;\pi_{i}\geq 0,\;1\leq i\leq k,\;\sum_{i=1}^{k}\pi_{i}=1\right\}$, $X∼M_{k}\left(n;\pi \right)$ in short. Notice that, if $\pi \in \Delta_{0k}$, then some components of π may equal 0, implying that some cell frequencies can be equal to zero, even for large samples. In many instances, it is assumed that π belongs to a parametric family $\pi \in P=\left\{P\left(\theta \right)={\left(p_{1}\left(\theta \right),\ldots ,p_{k}\left(\theta \right)\right)}^{t},\;\theta \in \Theta \right\}\subset \Delta_{k}=\left\{{\left(\pi_{1},\ldots ,\pi_{k}\right)}^{t}:\;\pi_{i}\;>\;0,\;1\leq i\leq k,\;\sum_{i=1}^{k}\pi_{i}=1\right\}$, where $\Theta \subseteq R^{s}$, k−s−1>0 and $p_{1}\left(\cdot \right)$, …, $p_{k}\left(\cdot \right)$ are known real functions.

When it is assumed that π∈P, π is usually estimated through $P\left(\hat{\theta }\right)={\left(p_{1}\left(\hat{\theta }\right),\ldots ,p_{k}\left(\hat{\theta }\right)\right)}^{t}$ for some estimator $\hat{\theta }$ of θ. A common choice for $\hat{\theta }$ is the maximum likelihood estimator (MLE), which is known to have good asymptotic properties. Basu and Sarkar [2] and Morales et al. [3] have shown that these properties are shared by a larger class of estimators: the minimum ϕ-divergence estimators (MϕE). This class includes MLEs as a particular case. However, as illustrated in Mandal et al. [4], the finite sample performance of these estimators can be improved by modifying the weight that each ϕ-divergence assigns to the empty cells. The resulting estimator is called the minimum penalized ϕ-divergence estimator (MPϕE). Moreover, Mandal et al. [4] have shown that such estimators have the same asymptotic properties as the MϕEs. Specifically, they are strongly consistent and, conveniently normalized, asymptotically normal. To derive these asymptotic properties, it is assumed that the probability model is correctly specified, that is to say, that we are sure about π∈P.

If the parametric model is not correctly specified, Jiménez-Gamero et al. [5] have shown that, under certain assumptions, the MϕEs still have a well defined limit, and, conveniently normalized, they are asymptotically normal. For the MLE, these results were known from those in [6]. Because, as argued before, the use of penalized ϕ-divergences may lead to better performance of the resulting estimators, the aim of this piece of research is to investigate the asymptotic properties of the MPϕEs under model misspecification. If the model considered is true, we obtain as a particular case the results in [4].

The usefulness of the results obtained is illustrated by applying them to the problem of testing goodness-of-fit to the parametric family P,

$$H_{0}:\;\pi \in P,$$

against the alternative

$$H_{1}:\;\pi \notin P,$$

using as a test statistic a penalized $\phi_{1}$-divergence between a nonparametric estimator of π, the relative frequencies, and a parametric estimator of π, obtained by assuming that the null hypothesis is true, $P(\hat{\theta })$, $\hat{\theta }$ being an MP$\phi_{2}$E. Here, $\phi_{1}$ and $\phi_{2}$ may differ. The convenience of using this type of test statistics is justified in Mandal et al. [7]. Although these authors show that, under $H_{0}$, such test statistics are asymptotically distribution free, the asymptotic approximation to the null distribution of the test statistics in this class is rather poor. Some numerical examples illustrate this unsatisfactory behavior of the asymptotic approximation. By using the fact that the MPϕE always converges to a well-defined limit, whether the model in $H_{0}$ is true or not, we prove that the bootstrap consistently estimates the null distribution of these test statistics. We then retake the previously cited numerical examples to exemplify the usefulness of the bootstrap approximation which, despite the demand for more computing time, is more accurate than that yielded by the asymptotic null distribution for small and moderate sample sizes.

The rest of the paper is organized as follows. Section 2 studies certain asymptotic properties of MP$\phi_{2}$Es; specifically, conditions are given for the strong consistency and asymptotic normality. Section 3 uses such results to prove that a parametric bootstrap provides a consistent estimator to the null distribution of test statistics based on penalized ϕ-divergences for testing $H_{0}$. Section 4 displays an application of the results obtained in the context of a classification work in a cover land map.

Before ending this section we introduce some notation: all limits in this paper are taken when n→∞; $\overset{L}{\to }$ denotes convergence in distribution; $\overset{P}{\to }$ denotes convergence in probability; $\overset{a.s.}{\to }$ denotes the almost sure convergence; let $\left\{A_{n}\right\}$ be a sequence of random variables and let ϵ∈R, then $A_{n}=O_{P}\left(n^{-\epsilon }\right)$ means that $n^{\epsilon }A_{n}$ is bounded in probability, $A_{n}=o_{P}\left(n^{-\epsilon }\right)$ means that $n^{\epsilon }A_{n}\overset{P}{\to }0$, and $A_{n}=o\left(n^{-\epsilon }\right)$ means that $n^{\epsilon }A_{n}\overset{a.s.}{\to }0$; $N_{k}\left(\mu ,\Sigma \right)$ denotes the *k*-variate normal law with mean μ and variance matrix Σ; all vectors are column vectors; the superscript ${}^{t}$ denotes transpose; if $x\in R^{k}$, with $x^{t}=\left(x_{1},\ldots ,x_{k}\right)$, then Diag(x) is the k×k diagonal matrix whose (i,i) entry is $x_{i}$, 1≤i≤k, and

$$\Sigma_{x}=Diag\left(x\right)-xx^{t};$$

$I_{k}$ denotes the k×k identity matrix; to simplify notation, all 0s appearing in the paper represent vectors of the appropriate dimension.

## 2. Some Asymptotic Properties of MPϕEs

Let $X∼M_{k}\left(n;\pi \right)$, with $\pi \in \Delta_{0k}$, and let $\hat{\pi }={\left({\hat{\pi }}_{1},{\hat{\pi }}_{2},\ldots ,{\hat{\pi }}_{k}\right)}^{t}$ be the vector of relative frequencies,

(1) $${\hat{\pi }}_{i}=\frac{X_{i}}{n},\;1\leq i\leq k.$$

Let P be a parametric model satisfying Assumption 1 below.

**Assumption** **1.** *$P=\left\{P\left(\theta \right)={\left(p_{1}\left(\theta \right),\ldots ,p_{k}\left(\theta \right)\right)}^{t},\;\theta \in \Theta \right\}\subset \Delta_{k}$, where $\Theta \subseteq R^{s}$, k−s−1>0 and $p_{1}\left(.\right)$, …, $p_{k}\left(.\right):\Theta ⟶R$ are known twice continuously differentiable in intΘ functions.*

Let ϕ:[0,∞)→R∪{∞} be a continuous convex function. For arbitrary $Q={\left(q_{1},\ldots ,q_{k}\right)}^{t}\in \Delta_{0k}$ and $P={\left(p_{1},\ldots ,p_{k}\right)}^{t}\in \Delta_{k}$, the ϕ-divergence between *Q* and *P* is defined by (Csiszár [8])

$$D_{\phi }\left(Q,P\right)=\sum_{i=1}^{k}p_{i}\phi \left(q_{i}/p_{i}\right).$$

Note that

$$D_{\phi }\left(Q,P\right)=\sum_{i/q_{i}>0}p_{i}\phi \left(q_{i}/p_{i}\right)+\phi \left(0\right)\sum_{i/q_{i}=0}p_{i}.$$

The penalized ϕ-divergence for the tuning parameter *h* between *Q* and *P* is defined from the above expression by replacing ϕ(0) with *h* as follows (see Mandal et al. [4]):

$$D_{\phi ,h}\left(Q,P\right)=\sum_{i/q_{i}>0}p_{i}\phi \left(q_{i}/p_{i}\right)+h\sum_{i/q_{i}=0}p_{i}.$$

If

$${\hat{\theta }}_{\phi ,h}=\arg\min_{\theta }D_{\phi ,h}\left(\hat{\pi },P\left(\theta \right)\right),$$

then ${\hat{\theta }}_{\phi ,h}$ is called the MPϕE of θ.

In order to study some of the properties of ${\hat{\theta }}_{\phi ,h}$, we will assume that ϕ satisfies Assumption 2 below.

**Assumption** **2.** *ϕ:[0,∞)→R is a strictly convex function, twice continuously differentiable in (0,∞).*

Assumption 2 is assumed when dealing with estimators based on minimum divergence, since it lets us take Taylor series expansions of $D_{\phi }\left(\hat{\pi },P\left(\theta \right)\right)$, which is useful to derive asymptotic properties of the MϕEs. For example, Section 3 of Lindsay [9] assumes that the function ϕ (he calls *G* what we call ϕ) is a thrice differentiable function (which is stronger than Assumption 2); Theorem 3 in Morales et al. [3] requires, among other conditions, ϕ to meet Assumption 2 to derive the consistency and asymptotic normality of MϕEs.

Assumption 2 is also assumed in Mandal et al. [4] (they call *G* what we call ϕ) to study the consistency and asymptotic normality of MPϕEs. Specifically, these authors show that, if π∈P and $\theta_{0}$ is the true parameter value, then, under suitable regularity conditions including Assumption 2, the MPϕE is consistent for $\theta_{0}$, and $\sqrt{n}\left({\hat{\theta }}_{\phi ,h}-\theta_{0}\right)$ is asymptotically normal with a mean of 0 and a variance matrix equal to the inverse of the information matrix.

Next we will only assume that $\pi \in \Delta_{0k}$, that is, the assumption that π∈P is dropped. In this context, we prove that the MPϕE is consistent for $\theta_{0}$, where now $\theta_{0}$ is the parameter vector that minimizes $D_{\phi ,h}\left(\pi ,P\left(\theta \right)\right)$, that is to say, $\theta_{0}=\arg\min_{\theta }D_{\phi ,h}\left(\pi ,P\left(\theta \right)\right)$. Note that $\theta_{0}$ also depends on ϕ and *h*, so to be rigorous we should denote it by $\theta_{0,\phi ,h}$, but to simplify notation we will simply denote it as $\theta_{0}$. We also show that $\sqrt{n}\left({\hat{\theta }}_{\phi ,h}-\theta_{0}\right)$ is asymptotically normal with a mean of 0. With this aim, we will also assume the following.

**Assumption** **3.** *$D_{\phi ,h}\left(\pi ,P\left(\theta \right)\right)$ has a unique minimum at $\theta_{0}\in int\Theta$.*

Assumption 3 is assumed in papers on estimators based on minimum divergence estimation. For example, it is Assumption A3(b) in [6], which states, that it is the fundamental identification condition for quasi-maximum likelihood estimators to have a well-defined limit; and it is contained in Assumptions 7 and 9 in [10], required for minimum chi-square estimators to have a well-defined limit; it also coincides with Assumption 30 in [9], imposed for the same reason.

Let $\theta_{0}$ be as defined in Assumption 3. Then $P(\theta_{0})$ is the (ϕ,h)-projection of π on P. Section 3 in [11] shows that Assumption 3 holds for two-way tables when P is the uniform association model, so the (ϕ,h)-projection always exists for such model. Nevertheless, this projection may not exist, or may not be defined uniquely. See Example 2 in [12] for an instance where there is no unique minimum (because although Θ is that example is convex, the family {P(θ),θ∈Θ} is not convex, so the uniqueness of the projection is not guaranteed). Let $\Delta_{k}\left(\phi ,P,h\right)=\left\{\pi \in \Delta_{0k}\;\mathrm{such}\;\mathrm{that}\;\mathrm{Assumption}3\mathrm{holds}\right\}$.

From now on, we will assume that the components of π are sorted so that $\pi_{1},\ldots ,\pi_{m}>0$, and $\pi_{m+1}=\ldots =\pi_{k}=0$, for some 1<m≤k, where, if m=k, then it is understood that all components of π are positive. We will write $\pi^{+}={\left(\pi_{1},\ldots ,\pi_{m}\right)}^{t}$ and ${\hat{\pi }}^{+}={\left({\hat{\pi }}_{1},\ldots ,{\hat{\pi }}_{m}\right)}^{t}$. The next result shows the strong consistency and asymptotic normality of the MPϕE.

**Theorem** **1.** *Let P be a parametric family satisfying Assumption 1. Let ϕ be a real function satisfying Assumption 2. Let $X∼M_{k}\left(n;\pi \right)$ with $\pi \in \Delta_{k}\left(\phi ,P,h\right)$. Then*

*(a)*  *${\hat{\theta }}_{\phi ,h}\overset{a.s.}{⟶}\theta_{0}$.*
*(b)*  *$\sqrt{n}\left(\begin{array}{c} {\hat{\pi }}^{+}-\pi^{+}\\ {\hat{\theta }}_{\phi ,h}-\theta_{0} \end{array}\right)\overset{L}{⟶}N_{m+s}\left(0,A\Sigma_{\pi^{+}}A^{t}\right),$ where $A^{t}=\left(I_{m},\;\;G^{t}\right)$ and G is defined in Equation* (7)*. In particular,*
  (2) $$\sqrt{n}\left({\hat{\theta }}_{\phi ,h}-\theta_{0}\right)\overset{L}{⟶}N_{s}\left(0,G\Sigma_{\pi^{+}}G^{t}\right)$$
*(c)*  *$\sqrt{n}\left(\begin{array}{c} {\hat{\pi }}^{+}-\pi^{+}\\ P\left({\hat{\theta }}_{\phi ,h}\right)-P\left(\theta_{0}\right) \end{array}\right)\overset{L}{⟶}N_{2m}\left(0,B\Sigma_{\pi^{+}}B^{t}\right)$, where $B^{t}=\left(I_{m},\;\;G^{t}D_{1}\left(P\left(\theta_{0}\right)\right)\right)$, with $D_{1}\left(P\left(\theta \right)\right)$ defined in Equation* (8).

**Remark** **1.** *Observe that, if m=k, then the penalization has no effect asymptotically; by contrast, if m<k, then the presence of the tuning parameter h influences the covariance matrix of the asymptotic law of $\sqrt{n}\left({\hat{\theta }}_{\phi ,h}-\theta_{0}\right)$ and $\sqrt{n}\left(P\left({\hat{\theta }}_{\phi ,h}\right)-P\left(\theta_{0}\right)\right)$.*

**Remark** **2.** *If π∈P, we obtain as a particular case the results in Mandal et al.* [4]. *Our conditions are weaker than those in* [4]. *The reason is that they allow an infinite number of categories, while we are assuming that such a number is finite, k. Therefore, when the number of categories is finite, the assumptions in* [4] *for the consistency and asymptotic normality of the MPϕE can be weakened.*

As a consequence of Theorem 1, the following corollary gives the asymptotic behavior of $D_{\phi_{1},h_{1}}\left(\hat{\pi },P\left({\hat{\theta }}_{\phi_{2},h_{2}}\right)\right)$, for arbitrary $\phi_{1}$, $\phi_{2}$, and $h_{1}$, $h_{2}$, that may or may not coincide. Part (a) of Corollary 1, which assumes that the model P is correctly specified, has been previously proven in [7]. It is included here for the sake of completeness. Part (b), which describes the limit in law under alternatives is, to the best of our knowledge, new.

**Corollary** **1.** *Let P be a parametric family satisfying Assumption 1. Let $\phi_{1}$ and $\phi_{2}$ be two real functions satisfying Assumption 2. Let $X∼M_{k}\left(n;\pi \right)$ with $\pi \in \Delta_{k}\left(\phi ,P,h\right)$.*

*(a)*  *For π∈P,*
  $$T=\frac{2n}{\phi_{1}^{\prime \prime }\left(1\right)}\left\{D_{\phi_{1},h_{1}}\left(\hat{\pi },P\left({\hat{\theta }}_{\phi_{2},h_{2}}\right)\right)-\phi_{1}\left(1\right)\right\}\overset{L}{⟶}\chi_{k-s-1}^{2}.$$
*(b)*  *For $\pi \in \Delta_{k}\left(\phi_{2},P,h_{2}\right)-P$, let $\theta_{0}=\arg\min_{\theta }D_{\phi_{2},h_{2}}\left(\pi ,P\left(\theta \right)\right)$. Then*
  $$W=\sqrt{n}\left\{D_{\phi_{1},h_{1}}\left(\hat{\pi },P\left({\hat{\theta }}_{\phi_{2},h_{2}}\right)\right)-D_{\phi_{1},h_{1}}\left(\pi ,P\left(\theta_{0}\right)\right)\right\}\overset{L}{⟶}N\left(0,\varrho^{2}\right)$$
  *where $\varrho^{2}=a^{t}B\Sigma_{\pi }B^{t}a$, with B, as defined in Theorem 1 with $\phi =\phi_{2}$ and $h=h_{2}$,*
  $$a^{t}=\left(\phi_{1}^{\prime }\left(\frac{\pi_{1}}{p_{1}\left(\theta_{0}\right)}\right),\ldots ,\phi_{1}^{\prime }\left(\frac{\pi_{m}}{p_{m}\left(\theta_{0}\right)}\right),v_{1},\ldots ,v_{m},\underset{k-m\mathrm{times}}{\underset{︸}{h_{1},\ldots ,h_{1}}}\right),$$
  *and $v_{i}$, 1≤i≤m, are as defined in Equation* (5)* with $\phi =\phi_{1}$ and $h=h_{1}$.*

**Remark** **3.** *If π∈P, the asymptotic behavior of the statistic T does not depend either on $\phi_{1}$, $\phi_{2}$, or on $h_{1}$, $h_{2}$. In fact, the asymptotic law of T is the same as if non-penalized divergences were used.*

**Remark** **4.** *When $\pi \in \Delta_{k}\left(\phi_{2},P,h_{2}\right)-P$, if m=k, then the asymptotic distribution of W does not depend on $h_{1}$, $h_{2}$; by contrast, if m<k, then the asymptotic distribution of W does depend on $h_{1}$ and $h_{2}$.*

**Remark** **5.** *(Properties of the asymptotic test) As a consequence of Corollary 1(a), we have that for testing $H_{0}$ vs. $H_{1}$, the test that rejects the null hypothesis when $T\geq \chi_{k-s-1,1-\alpha }^{2}$ is asymptotically correct, in the sense that $P_{0}\left(T\geq \chi_{k-s-1,1-\alpha }^{2}\right)\to \alpha$, where $\chi_{k-s-1,1-\alpha }^{2}$ stands for the 1−α percentile of the $\chi_{k-s-1}^{2}$ distribution and $P_{0}$ stands for the probability when the null hypothesis is true. From Corollary 1(b), it follows that such a test is consistent against fixed alternatives $\pi \in \Delta_{k}\left(\phi_{2},P,h_{2}\right)-P$, in the sense that $P(T\geq \chi_{k-s-1,1-\alpha }^{2})\to 1$.*

## 3. Application to Bootstrapping Goodness-Of-Fit Tests

As observed in Remark 5, the test that rejects $H_{0}$ when $T\geq \chi_{k-s-1,1-\alpha }^{2}$ is asymptotically correct and consistent against fixed alternatives. Nevertheless, the $\chi^{2}$ approximation to the null distribution of the test statistic is rather poor. Next we illustrate this fact with three examples. The last one is motivated by a real data set application in Section 4. All computations have been performed using programs written in the R language [13].

**Example** **1.** *Let $X∼M_{3}\left(n;\pi \right)$, with π∈P so that*

$$p_{1}\left(\theta \right)=\frac{1}{3}-\theta ,\;\;p_{2}\left(\theta \right)=\frac{2}{3}-\theta ,\;\;p_{3}\left(\theta \right)=2\theta ,\;\;0<\theta <1/3.$$

The problem of testing goodness-of-fit to this family is dealt with by considering as test statistic a penalized $\phi_{1}$-divergence and an MP$\phi_{2}$E, with $\phi_{1}$ and $\phi_{2}$, two members of the power-divergence family, defined as follows:

$$PD_{\lambda }\left(x\right)=\frac{1}{\lambda (\lambda +1)}\left(x^{\left(\lambda +1\right)}-x-\lambda \left(x-1\right)\right),\lambda \neq 0,-1,$$

$PD_{0}\left(x\right)=x\log\left(x\right)-x+1$, for λ=0, and $PD_{-1}\left(x\right)=-\log\left(x\right)+x-1$, for λ=−1. We thank an anonymous referee for pointing out that the power divergence family is also known as the α-divergence family (see, for example, Section 4 of Amari [14]).

In order to evaluate the performance of the $\chi^{2}$ approximation to the null distribution of *T*, we carried out an extensive simulation experiment. As a previous part of the simulation experiment, we evaluated the possible effect of the tuning parameter $h_{2}$ on the accuracy of the MP$\phi_{2}$E. For this goal, we generated 10,000 samples of size 200 from the parametric family with θ=0.3333, and calculated the MP$\phi_{2}$E with $h_{2}=0.5,1,2,5,10$ and $\phi_{2}=PD_{-2}$, which correspond to the modified chi-square test statistic (see, for example, [1], p. 114). We calculated the root mean square deviation (RMSD) of the resulting estimations,

$$RMSD=\sqrt{\frac{\sum_{i=1}^{10,000}{\left({\hat{\theta }}_{-2,h_{2}}-\theta \right)}^{2}}{10,000}},$$

obtaining 0.00156, 0.00128, 0.00128, 0.00128, and 0.00128, respectively. According to these results, there are rather small differences in the performance of the MP$\phi_{2}$E for the values of $h_{2}$ considered. Because of this, we fixed $\phi_{2}=PD_{-2}$ and $h_{2}=0.5,1,2$.

Next, to study the goodness of the asymptotic approximation, we generated 10,000 samples of size n=100 from the parametric family with θ=0.3333, and calculated the test statistic *T* with $h_{1}=h_{2}=0.5$ and $\phi_{1}\left(x\right)=\phi_{2}\left(x\right)=PD_{-2}\left(x\right)$, as well as the associated *p*-values corresponding to the asymptotic null distribution. We then computed the fraction of these *p*-values, which are less than or equal to the nominal values α=0.05,0.10 (top and below in tables). This experiment was repeated for n=150,200, $h_{1}=h_{2}=1,2$, $\phi_{1}=PD_{1}$ (which corresponds to the chi-square test statistic) and $\phi_{1}=PD_{2}$. Table 1 shows the results obtained. We also considered the case $h_{1}\neq h_{2}$, obtaining quite close outcomes. Table 2 displays the results obtained for n=200 and $\phi_{1}=\phi_{2}=PD_{-2}$. Looking at these tables, we conclude that the asymptotic null distribution does not provide an accurate estimation of the null distribution of *T* since the type I error probabilities are much greater than the nominal values, 0.05 and 0.10. Therefore, other approximations of the null distribution should be studied.

**Example** **2.** *Let $X∼M_{3}\left(n;\pi \right)$, with π∈P so that*

$$p_{1}\left(\theta \right)=0.5-2\theta ,\;\;p_{2}\left(\theta \right)=0.5+\theta ,\;\;p_{3}\left(\theta \right)=\theta ,\;\;0<\theta <1/4.$$

We repeated the simulation schedule described in Example 1 for this law with θ=0.24. Table 3 and Table 4 report the obtained results. In contrast to the results for Example 1, where the asymptotic approximation gives a rather liberal test, in this case the resulting test is very conservative. Therefore, we again conclude that the asymptotic null distribution does not provide an accurate estimation of the null distribution of *T*.

**Example** **3.** *Let $X∼M_{4}\left(n;\pi \right)$, with π∈P so that*

(3) $$p_{1}\left(\theta \right)=\theta^{2},\;\;p_{2}\left(\theta \right)=\theta \left(1-\theta \right),\;\;p_{3}\left(\theta \right)=\theta \left(1-\theta \right),\;\;p_{4}\left(\theta \right)={\left(1-\theta \right)}^{2},\;\;0<\theta <1.$$

We repeated the simulation schedule described in Example 1 for this law with θ=0.8. Table 5 and Table 6 report the results obtained. Looking at these tables, we see that the test based on asymptotic approximation is liberal, and conclude, as in the previous examples, that other approximations of the null distribution should be considered.

The reason for the unsatisfactory results in the three examples is that the asymptotic approximation requires unaffordably large sample sizes when some cells have extremely small probabilities, which provoke the presence of zero cell frequencies. To appreciate this fact, notice that Example 1 requires n>30,000 to obtain expected cell frequencies greater than 10.

Motivated by these examples, the aim of this section is to study another way of approximating the null distribution of *T*, the bootstrap. The null bootstrap distribution of *T* is the conditional distribution of

$$T^{*}=\frac{2n}{\phi_{1}^{\prime \prime }\left(1\right)}\left\{D_{\phi_{1},h_{1}}\left({\hat{\pi }}^{*},P\left({\hat{\theta }}_{\phi_{2},h_{2}}^{*}\right)\right)-\phi_{1}\left(1\right)\right\},$$

given $(X_{1},\ldots ,X_{k}),$ where ${\hat{\pi }}^{*}$ is defined as $\hat{\pi }$ with $\left(X_{1},\ldots ,X_{k}\right)$ replaced by $\left(X_{1}^{*},\ldots ,X_{k}^{*}\right)∼M_{k}\left(n;P\left({\hat{\theta }}_{\phi_{2},h_{2}}\right)\right)$, and ${\hat{\theta }}_{\phi_{2},h_{2}}^{*}=\arg\min_{\theta }D_{\phi_{2},h_{2}}\left({\hat{\pi }}^{*},P\left(\theta \right)\right)$.

Let $P_{*}$ denote the bootstrap conditional probability law, given $\left(X_{1},\ldots ,X_{k}\right)$. The next theorem gives the weak limit of $T^{*}$.

**Theorem** **2.** *Let P be a parametric family satisfying Assumption 1. Let $\phi_{1}$ and $\phi_{2}$ be two real functions satisfying Assumption 2. Let $X∼M_{k}\left(n;\pi \right)$ with $\pi \in \Delta_{k}\left(\phi ,P,h\right)$. Then*

$$\underset{x}{\sup}\left|P_{*}\left(T^{*}\leq x\right)-P\left(Y\leq x\right)\right|\overset{P}{⟶}0$$

*where $Y∼\chi_{k-s-1}^{2}$.*

Recall that, from Corollary 1(a), when $H_{0}$ is true, the test statistic *T* converges in law to a $\chi_{k-s-1}^{2}$ law. Thus, the result in Theorem 2 implies the consistency of the null bootstrap distribution of *T* as an estimator of the null distribution of *T*. It is important to remark that the result in Theorem 2 holds whether $H_{0}$ is true or not, that is, the bootstrap properly estimates the null distribution, even if the available data does not obey the law in the null hypothesis. This is due to the fact that, under the assumed conditions, the MPϕE always converges to a well-defined limit.

**Remark** **6.** Properties of the Bootstrap Test. Similarly to Remark 5, as a consequence of Corollary 1(a) and Theorem 2, we have that, for testing $H_{0}$ vs. $H_{1}$, the test that rejects the null hypothesis when $T\geq T_{1-\alpha }^{*}$ is asymptotically correct, in the sense that $P_{0}\left(T\geq T_{1-\alpha }^{*}\right)\to \alpha$, where $T_{1-\alpha }^{*}$ stands for the 1−α percentile of the bootstrap distribution of T. From Corollary 1(b) and Theorem 2, it follows that such a test is consistent against fixed alternatives $\pi \in \Delta_{k}\left(\phi_{2},P,h_{2}\right)-P$, in the sense that $P(T\geq T_{1-\alpha }^{*})\to 1$.

In practice, the bootstrap *p*-value must be approximated by simulation as follows:

1. Calculate the observed value of the test statistic for the available data $\left(X_{1},\ldots ,X_{k}\right)$, $T_{obs}$.
2. Generate *B* bootstrap samples $\left(X_{1}^{b*},\ldots ,X_{k}^{b*}\right)∼M_{k}\left(n;P\left({\hat{\theta }}_{\phi_{2},h_{2}}\right)\right)$, b=1,⋯,B, and calculate the test statistic for each bootstrap sample obtaining $T^{*b}$, b=1,⋯,B.
3. Approximate the *p*-value by means of the expression
  $${\hat{p}}_{boot}=\frac{\mathrm{card}\{b:T_{b}^{*b}\geq T_{obs}\}}{B}.$$

For the numerical experiments previously described, whose results are displayed in Table 1, Table 2, Table 3, Table 4, Table 5 and Table 6, we also calculated the bootstrap *p*-values. This was done by generating B=1000 bootstrap samples to approximate each *p*-value, and calculating the fraction of these *p*-values, which are less than or equal to 0.05 and 0.10 (top and bottom in the tables). Table 7, Table 8, Table 9, Table 10, Table 11 and Table 12 display the estimated type I error probabilities obtained by using the bootstrap approximation as well as those obtained with the asymptotic approximation (bootstrap, B, and asymptotic, A, in the tables) taken from Table 1, Table 2, Table 3, Table 4, Table 5 and Table 6 in order to facilitate the comparison between them. Looking at Table 7, Table 8, Table 9, Table 10, Table 11 and Table 12, we conclude that the bootstrap approximation is superior to the asymptotic one for small and moderate sample sizes, since in all cases the bootstrap type I error probabilities were closer to the nominal values than those obtained using the asymptotic null distribution. This superior performance of the bootstrap null distribution estimator has been noticed in other inferential problems, where ϕ-divergences are used as test statistics (see, for example, [5,12,15,16]).

## 4. Application to the Evaluation of the Thematic Classification in Global Land Cover Maps

This section displays the results of an application of our proposal to two real data sets related to the thematic quality assessment of a global land cover (GLC) map. The data comprise the results of two thematic classifications of the land cover category “Evergreen Broadleaf Trees” (EBL) and summarize the number of sample units correctly classified in this class, and the number of confusions with other land cover classes: “Deciduous Broadleaf Trees” (DBL), “Evergreeen Needleleaf Trees” (ENL), and “Urban/Built Up” (U). The results of these two classifications were collected from two different global land cover maps: the Globcover map and the LC-CCI map (see Tsendbazar et al. [17] for additional details) and they are displayed in Table 13.

Parametric specifications of the multinomial vector of probabilities are quite attractive since they describe in a concise way the classification pattern. Because of this, given the similarity between the two observed classifications in Table 13, we are interested in the search of a parametric model suitable to depict the thematic accuracy of this class in both GLC maps. For this purpose, we consider the parametric family in Equation (3) of Example 3. The presence of a zero cell frequency in each data set leads us to consider a penalized ϕ-divergence as a test statistic for testing goodness-of-fit to such a parametric family.

Table 14 displays the observed values of the test statistic *T* and the associated bootstrap *p*-values for the goodness-of-fit test with respect to the parametric family in Equation (3) for the two observed classifications of the EBL class in Table 13. Looking at this table, it can be concluded that the null hypothesis cannot be rejected in both cases. Therefore, the parametric model in Equation (3) provides an adequate description of the thematic classification of the EBL class.

## 5. Proofs

Notice that

$$\begin{array}{ccc} D_{\phi ,h}\left(\pi ,P\left(\theta \right)\right) & = & \sum_{i=1}^{m}p_{i}\left(\theta \right)\phi \left(\frac{\pi_{i}}{p_{i}\left(\theta \right)}\right)+h\sum_{i=m+1}^{k}p_{i}\left(\theta \right)\\ & = & hI\left(m<k\right)+\sum_{i=1}^{m}p_{i}\left(\theta \right)\phi_{h}\left(\frac{\pi_{i}}{p_{i}\left(\theta \right)}\right) \end{array}$$

where I stands for the indicator function, $\phi_{h}\left(x\right)=\phi \left(x\right)-h$, if m<k, and $\phi_{h}\left(x\right)=\phi \left(x\right)$, if m=k. Let

$$D_{\phi ,h}^{+}\left(\pi ,P\left(\theta \right)\right)=\sum_{i=1}^{m}p_{i}\left(\theta \right)\phi_{h}\left(\frac{\pi_{i}}{p_{i}\left(\theta \right)}\right).$$

Clearly,

$$\arg\min_{\theta }D_{\phi ,h}\left(\hat{\pi },P\left(\theta \right)\right)=\arg\min_{\theta }D_{\phi ,h}^{+}\left(\hat{\pi },P\left(\theta \right)\right).$$

Note that, if Assumptions 1 and 2 hold, then Assumption 3 implies that

(4) $$\frac{\partial }{\partial \theta }D_{\phi }^{+}\left(\pi ,P\left(\theta_{0}\right)\right)=\sum_{i=1}^{m}\frac{\partial }{\partial \theta }p_{i}\left(\theta_{0}\right)v_{i}=0$$

where

(5) $$v_{i}=\phi \left(\frac{\pi_{i}}{p_{i}\left(\theta_{0}\right)}\right)-\frac{\pi_{i}}{p_{i}\left(\theta_{0}\right)}\phi^{\prime }\left(\frac{\pi_{1}}{p_{i}\left(\theta_{0}\right)}\right)-hI\left(m<k\right)$$

1≤i≤m, and $\phi^{\prime }\left(x\right)=\frac{\partial }{\partial x}\phi \left(x\right)$. The s×s matrix

(6) $$D_{2}=\frac{\partial^{2}}{\partial \theta \partial \theta^{t}}D_{\phi }^{+}\left(\pi ,P\left(\theta_{0}\right)\right)=\sum_{i=1}^{m}\frac{\partial^{2}}{\partial \theta \partial \theta^{t}}p_{i}\left(\theta_{0}\right)v_{i}+\sum_{i=1}^{m}\frac{\partial }{\partial \theta }p_{i}\left(\theta_{0}\right)\frac{\partial }{\partial \theta }p_{i}{\left(\theta_{0}\right)}^{t}w_{i}$$

is positive definite, where

$$w_{i}=\frac{\pi_{i}^{2}}{p_{i}^{3}\left(\theta_{0}\right)}\phi^{\prime \prime }\left(\frac{\pi_{i}}{p_{i}\left(\theta_{0}\right)}\right),$$

1≤i≤m, and $\phi^{\prime \prime }\left(x\right)=\frac{\partial^{2}}{\partial x^{2}}\phi \left(x\right)$. Therefore, by the Implicit Function Theorem (see, for example, Dieudonne [18], p. 272), there is an open neighborhood $U\subseteq {\left(0,1\right)}^{m}$ of $\pi^{+}$ and *s* unique functions, $g_{i}:U\to R$, 1≤i≤s, so that

(i) ${\hat{\theta }}_{\phi }={\left(g_{1}\left({\hat{\pi }}^{+}\right),\ldots ,g_{s}\left({\hat{\pi }}^{+}\right)\right)}^{t}$, $\forall \;n\geq n_{0}$, for some $n_{0}\in N$;
(ii) $\theta_{0}={\left(g_{1}\left(\pi^{+}\right),\ldots ,g_{s}\left(\pi^{+}\right)\right)}^{t}$;
(iii) $g={\left(g_{1},\ldots ,g_{s}\right)}^{t}$ is continuously differentiable in *U* and the s×m Jacobian matrix of *g* at $\left(\pi_{1},\ldots ,\pi_{m}\right)$ is given by
  (7) $$G=D_{2}^{-1}D_{1}\left(P\left(\theta_{0}\right)\right)Diag\left(\varpi \right)$$
  where
  (8) $$D_{1}\left(P\left(\theta \right)\right)=\left(\frac{\partial }{\partial \theta }p_{1}\left(\theta \right),\ldots ,\frac{\partial }{\partial \theta }p_{m}\left(\theta \right)\right),$$
  $\varpi ={\left(\varpi_{1},\ldots ,\varpi_{m}\right)}^{t}$,
  $$\varpi_{i}=\frac{\pi_{i}}{p_{i}^{2}\left(\theta_{0}\right)}\phi^{\prime \prime }\left(\frac{\pi_{i}}{p_{i}\left(\theta_{0}\right)}\right),$$
  and 1≤i≤m.

**Proof** **of** **Theorem** **1.** Part (a) follows from (i) and (ii) above and the fact that ${\hat{\pi }}^{+}\to \pi^{+}$ a.s. From (i)–(ii), and taking into account that $\sqrt{n}\left({\hat{\pi }}^{+}-\pi^{+}\right)$ is asymptotically normal, it follows that

(9) $${\hat{\theta }}_{\phi }=\theta_{0}+G\left(\pi ,P\left(\theta_{0}\right),\phi \right)\left(\hat{\pi }-\pi \right)+o_{P}\left(n^{-1/2}\right).$$

Parts (b) and (c) follow from Equation (9) and the asymptotic normality of $\sqrt{n}\left({\hat{\pi }}^{+}-\pi^{+}\right)$. ☐

**Proof** **of** **Corollary** **1.** Part (a) was shown in Theorem 5.1 in [7]. To prove (b), we first demonstrate that

(10) $$W=W_{0}+r_{n}$$

where

$$W_{0}=\sqrt{n}\left\{\sum_{j=1}^{m}p_{j}\left({\hat{\theta }}_{\phi_{2},h_{2}}\right)\phi_{1}\left(\frac{{\hat{\pi }}_{j}}{p_{j}\left({\hat{\theta }}_{\phi_{2},h_{2}}\right)}\right)+h_{1}\sum_{j=m+1}^{k}p_{j}\left({\hat{\theta }}_{\phi_{2},h_{2}}\right)-D_{\phi_{1},h_{1}}\left(\pi ,P\left(\theta_{0}\right)\right)\right\}+r_{n},$$

and $r_{n}=o_{P}\left(1\right)$. Notice that

$$\begin{array}{ccc} r_{n} & = & \sqrt{n}\left\{h_{1}-\phi_{1}\left(0\right)\right\}\sum_{j:{\hat{\pi }}_{j}=0,\;\pi_{j}>0}p_{j}\left({\hat{\theta }}_{\phi_{2},h_{2}}\right)\\ & = & \sqrt{n}\left\{h_{1}-\phi_{1}\left(0\right)\right\}\sum_{j=1}^{m}p_{j}\left({\hat{\theta }}_{\phi_{2},h_{2}}\right)I\left({\hat{\pi }}_{j}=0\right). \end{array}$$

Therefore,

$$0\leq E|r_{n}|\leq \sqrt{n}|h_{1}-\phi_{1}\left(0\right)|\sum_{j=1}^{m}P\left({\hat{\pi }}_{j}=0\right)=\sqrt{n}\left|h_{1}-\phi_{1}\left(0\right)\right|\sum_{j=1}^{m}{\left(1-\pi_{j}\right)}^{n}\to 0,$$

which implies $r_{n}=o_{P}\left(1\right)$. From Theorem 1 and Taylor expansion, it follows that $W_{0}\overset{L}{⟶}N\left(0,\varrho^{2}\right)$; hence, the result in part (b) is proven. ☐

**Proof** **of** **Theorem** **2.** The proof of Theorem 2 is parallel to that of Theorem 2 in [5], so we omit it. ☐

## Figures and Tables

**Table 1:** Type I error probabilities obtained using asymptotic approximation for Example 1 with θ=0.3333, $\phi_{1}=PD_{\lambda }$, λ∈{−2,1,2}, $\phi_{2}=PD_{-2}$, and $h_{1}=h_{2}\in \left\{0.5,1,2\right\}$.

	$\phi_{1}={\mathrm{PD}}_{-2}$			$\phi_{1}={\mathrm{PD}}_{1}$			$\phi_{1}={\mathrm{PD}}_{2}$		
	$h_{1}=h_{2}$			$h_{1}=h_{2}$			$h_{1}=h_{2}$		
*n*	0.5	1	2	0.5	1	2	0.5	1	2
100	0.996	0.996	0.998	0.995	0.997	0.996	0.995	0.997	0.997
	0.996	0.996	0.998	0.995	0.997	0.996	0.995	0.997	0.997
150	0.995	0.995	0.996	0.994	0.995	0.996	0.994	0.994	0.995
	0.995	0.995	0.996	0.994	0.995	0.996	0.994	0.994	0.995
200	0.992	0.993	0.994	0.992	0.994	0.991	0.993	0.993	0.994
	0.992	0.994	0.994	0.992	0.994	0.991	0.993	0.993	0.994

**Table 2:** Type I error probabilities obtained using asymptotic approximation for Example 1 with n=200, θ=0.3333, $\phi_{1}=\phi_{2}=PD_{-2}$, $h_{1}\neq h_{2}$, and $h_{1},h_{2}\in \left\{0.5,1,2\right\}$.

$\left(h_{1},h_{2}\right)$	(0.5, 1)	(1, 0.5)	(0.5, 2)	(2, 0.5)	(1, 2)	(2, 1)
	0.989	0.997	0.998	0.998	0.994	0.998
	0.999	0.997	0.998	0.998	0.994	0.999

**Table 3:** Type I error probabilities obtained using asymptotic approximation for Example 2 with θ=0.24, $\phi_{1}=PD_{\lambda }$, λ∈{−2,1,2}, $\phi_{2}=PD_{-2}$, and $h_{1}=h_{2}\in \left\{0.5,1,2\right\}$.

	$\phi_{1}={\mathrm{PD}}_{-2}$			$\phi_{1}={\mathrm{PD}}_{1}$			$\phi_{1}={\mathrm{PD}}_{2}$		
	$h_{1}=h_{2}$			$h_{1}=h_{2}$			$h_{1}=h_{2}$		
*n*	0.5	1	2	0.5	1	2	0.5	1	2
100	0.016	0.017	0.017	0.013	0.013	0.014	0.013	0.014	0.015
	0.034	0.036	0.036	0.031	0.030	0.031	0.030	0.033	0.033
150	0.018	0.019	0.017	0.014	0.014	0.014	0.013	0.015	0.016
	0.035	0.039	0.037	0.031	0.033	0.032	0.035	0.033	0.032
200	0.024	0.022	0.022	0.014	0.016	0.016	0.014	0.015	0.016
	0.043	0.042	0.040	0.032	0.034	0.032	0.032	0.035	0.033

**Table 4:** Type I error probabilities obtained using asymptotic approximation for Example 2 with n=200, θ=0.24, $\phi_{1}=\phi_{2}=PD_{-2}$, $h_{1}\neq h_{2}$, and $h_{1},h_{2}\in \left\{0.5,1,2\right\}$.

$\left(h_{1},h_{2}\right)$	(0.5, 1)	(1, 0.5)	(0.5, 2)	(2, 0.5)	(1, 2)	(2, 1)
	0.017	0.017	0.018	0.019	0.018	0.016
	0.035	0.033	0.035	0.040	0.036	0.034

**Table 5:** Type I error probabilities obtained using asymptotic approximation for Example 3 with θ=0.8, $\phi_{1}=PD_{\lambda }$, λ∈{−2,1,2}, $\phi_{2}=PD_{-2}$, and $h_{1}=h_{2}\in \left\{0.5,1,2\right\}$.

	$\phi_{1}={\mathrm{PD}}_{-2}$			$\phi_{1}={\mathrm{PD}}_{1}$			$\phi_{1}={\mathrm{PD}}_{2}$		
	$h_{1}=h_{2}$			$h_{1}=h_{2}$			$h_{1}=h_{2}$		
*n*	0.5	1	2	0.5	1	2	0.5	1	2
100	0.063	0.066	0.074	0.095	0.107	0.111	0.122	0.136	0.131
	0.122	0.120	0.125	0.157	0.165	0.161	0.181	0.190	0.182
150	0.063	0.064	0.066	0.083	0.082	0.084	0.099	0.105	0.100
	0.114	0.118	0.113	0.137	0.134	0.136	0.153	0.159	0.152
200	0.062	0.061	0.061	0.075	0.079	0.074	0.086	0.091	0.086
	0.111	0.111	0.115	0.129	0.137	0.123	0.145	0.148	0.144

**Table 6:** Type I error probabilities obtained using asymptotic approximation for Example 3 with n=200, θ=0.8, $\phi_{1}=\phi_{2}=PD_{-2}$, $h_{1}\neq h_{2}$, and $h_{1},h_{2}\in \left\{0.5,1,2\right\}$.

$\left(h_{1},h_{2}\right)$	(0.5, 1)	(1, 0.5)	(0.5, 2)	(2, 0.5)	(1, 2)	(2, 1)
	0.060	0.062	0.063	0.062	0.063	0.058
	0.108	0.114	0.113	0.112	0.113	0.109

**Table 7:** Asymptotic and bootstrap type I error probabilities for Example 1 with θ=0.3333, $\phi_{1}=PD_{\lambda }$, λ∈{−2,1,2}, $\phi_{2}=PD_{-2}$, $h_{1}=h_{2}\in \left\{0.5,1,2\right\}$.

	$h_{1}=h_{2}$	0.5		1		2	
$\phi_{1}$	*n*	B	A	B	A	B	A
$PD_{-2}$	100	0.051	0.996	0.048	0.996	0.048	0.998
		0.110	0.996	0.103	0.996	0.109	0.998
	150	0.055	0.995	0.050	0.995	0.056	0.996
		0.106	0.995	0.101	0.995	0.109	0.996
	200	0.053	0.992	0.053	0.993	0.056	0.994
		0.103	0.992	0.106	0.994	0.108	0.994
$PD_{1}$	100	0.057	0.995	0.056	0.997	0.055	0.996
		0.110	0.995	0.110	0.997	0.107	0.996
	150	0.054	0.994	0.052	0.995	0.055	0.996
		0.110	0.994	0.104	0.995	0.114	0.996
	200	0.055	0.992	0.051	0.994	0.052	0.991
		0.106	0.992	0.103	0.994	0.106	0.991
$PD_{2}$	100	0.055	0.995	0.056	0.997	0.054	0.997
		0.110	0.995	0.109	0.997	0.107	0.997
	150	0.054	0.994	0.055	0.994	0.056	0.995
		0.107	0.994	0.106	0.994	0.110	0.995
	200	0.054	0.993	0.053	0.993	0.055	0.994
		0.107	0.993	0.105	0.993	0.108	0.994

**Table 8:** Asymptotic and bootstrap type I error probabilities for Example 1 with n=200, θ=0.3333, $\phi_{1}=\phi_{2}=PD_{-2}$, $h_{1}\neq h_{2}$, and $h_{1},h_{2}\in \left\{0.5,1,2\right\}$.

$\left(h_{1},h_{2}\right)$	(0.5, 1)		(1, 0.5)		(0.5, 2)		(2, 0.5)		(1, 2)		(2, 1)	
	B	A	B	A	B	A	B	A	B	A	B	A
	0.061	0.989	0.050	0.997	0.059	0.996	0.042	0.998	0.044	0.994	0.063	0.998
	0.107	0.999	0.113	0.997	0.106	0.996	0.095	0.998	0.105	0.994	0.115	0.999

**Table 9:** Asymptotic and bootstrap type I error probabilities for Example 2 with θ=0.24, $\phi_{1}=PD_{\lambda }$, λ∈{−2,1,2}, $\phi_{2}=PD_{-2}$, and $h_{1}=h_{2}\in \left\{0.5,1,2\right\}$.

	$h_{1}=h_{2}$	0.5		1		2	
$\phi_{1}$	*n*	B	A	B	A	B	A
$PD_{-2}$	100	0.057	0.016	0.055	0.017	0.051	0.017
		0.111	0.034	0.110	0.036	0.102	0.036
	150	0.049	0.018	0.048	0.019	0.051	0.017
		0.097	0.035	0.103	0.039	0.101	0.036
	200	0.051	0.024	0.055	0.022	0.051	0.022
		0.099	0.043	0.102	0.042	0.099	0.040
$PD_{1}$	100	0.058	0.013	0.054	0.013	0.051	0.014
		0.114	0.031	0.113	0.030	0.106	0.031
	150	0.050	0.014	0.051	0.014	0.052	0.014
		0.098	0.031	0.103	0.031	0.100	0.032
	200	0.049	0.014	0.054	0.016	0.052	0.016
		0.099	0.032	0.104	0.034	0.099	0.032
$PD_{2}$	100	0.055	0.013	0.053	0.014	0.050	0.015
		0.110	0.030	0.108	0.033	0.104	0.033
	150	0.050	0.013	0.052	0.015	0.051	0.016
		0.097	0.032	0.103	0.033	0.098	0.032
	200	0.049	0.014	0.051	0.015	0.051	0.016
		0.100	0.032	0.102	0.035	0.098	0.033

**Table 10:** Asymptotic and bootstrap type I error probabilities for Example 2 with n=200, θ=0.24, $\phi_{1}=\phi_{2}=PD_{-2}$, $h_{1}\neq h_{2}$, and $h_{1},h_{2}\in \left\{0.5,1,2\right\}$.

$\left(h_{1},h_{2}\right)$	(0.5, 1)		(1, 0.5)		(0.5, 2)		(2, 0.5)		(1, 2)		(2, 1)	
	B	A	B	A	B	A	B	A	B	A	B	A
	0.048	0.017	0.051	0.017	0.052	0.018	0.053	0.019	0.050	0.018	0.049	0.016
	0.101	0.035	0.099	0.033	0.100	0.035	0.105	0.040	0.103	0.036	0.101	0.034

**Table 11:** Asymptotic and bootstrap type I error probabilities for Example 3 with θ=0.8, $\phi_{1}=PD_{\lambda }$, λ∈{−2,1,2}, $\phi_{2}=PD_{-2}$, and $h_{1}=h_{2}\in \left\{0.5,1,2\right\}$.

	$h_{1}=h_{2}$	0.5		1		2	
$\phi_{1}$	*n*	B	A	B	A	B	A
$PD_{-2}$	100	0.066	0.063	0.058	0.066	0.044	0.074
		0.119	0.122	0.101	0.120	0.086	0.125
	150	0.053	0.063	0.050	0.064	0.045	0.066
		0.098	0.114	0.095	0.118	0.093	0.113
	200	0.051	0.062	0.047	0.061	0.046	0.061
		0.099	0.111	0.096	0.111	0.100	0.115
$PD_{1}$	100	0.049	0.095	0.049	0.107	0.041	0.111
		0.103	0.157	0.098	0.065	0.084	0.161
	150	0.050	0.083	0.040	0.082	0.040	0.084
		0.098	0.137	0.090	0.134	0.087	0.136
	200	0.046	0.075	0.048	0.079	0.044	0.074
		0.095	0.129	0.102	0.137	0.092	0.123
$PD_{2}$	100	0.043	0.122	0.045	0.136	0.037	0.131
		0.099	0.181	0.046	0.190	0.077	0.182
	150	0.040	0.099	0.047	0.105	0.035	0.100
		0.041	0.153	0.093	0.159	0.081	0.152
	200	0.043	0.086	0.048	0.091	0.043	0.086
		0.092	0.145	0.097	0.148	0.090	0.144

**Table 12:** Asymptotic and bootstrap type I error probabilities for Example 3 with n=200, θ=0.8, $\phi_{1}=\phi_{2}=PD_{-2}$, $h_{1}\neq h_{2}$, and $h_{1},h_{2}\in \left\{0.5,1,2\right\}$.

$\left(h_{1},h_{2}\right)$	(0.5, 1)		(1, 0.5)		(0.5, 2)		(2, 0.5)		(1, 2)		(2, 1)	
	B	A	B	A	B	A	B	A	B	A	B	A
	0.047	0.060	0.048	0.062	0.051	0.063	0.049	0.062	0.048	0.063	0.044	0.058
	0.095	0.108	0.099	0.114	0.099	0.113	0.097	0.112	0.099	0.113	0.092	0.109

**Table 13:** Thematic classification of the Evergreen Broadleaf Trees (EBL) class.

		Globcover Map	LC-CCI Map
Classified Data	EBL	165	172
	DBL	13	5
	ENL	7	5
	U	0	0

**Table 14:** Results of the goodness-of-fit test applied to the thematic classification of the EBL class.

	Globcover Map				LC-CCI Map		
	${\hat{\theta }}_{-2,0.5}=0.9490$				${\hat{\theta }}_{-2,0.5}=0.9721$		
$\phi_{1}$	$PD_{-2}$	$PD_{1}$	$PD_{2}$		$PD_{-2}$	$PD_{1}$	$PD_{2}$
$T_{obs}$	2.3015	2.7618	3.0111		0.1432	0.1432	0.1433
${\hat{p}}_{boot}$	0.1700	0.2253	0.2926		0.9283	0.9200	0.9148
	${\hat{\theta }}_{-2,1}=0.9503$				${\hat{\theta }}_{-2,1}=0.9725$		
$T_{obs}$	2.7686	3.3752	3.6962		0.2821	0.2823	0.2826
${\hat{p}}_{boot}$	0.1801	0.2325	0.2671		0.8431	0.9162	0.9182
	${\hat{\theta }}_{-2,2}=0.9527$				${\hat{\theta }}_{-2,2}=0.9732$		
$T_{obs}$	3.6352	4.5400	5.0219		0.5492	0.5508	0.5514
${\hat{p}}_{boot}$	0.1300	0.2492	0.2584		0.7526	0.8144	0.8291

## Abbreviations

The following abbreviations are used in this manuscript:

MLE	maximum likelihood estimator
MϕE	minimum ϕ-divergence estimator
MPϕE	minimum penalized ϕ-divergence estimator
RMSD	root mean square deviation
B	bootstrap
A	asymptotic
GLC	global land cover
EBL	evergreen broadleaf trees
DBL	deciduous broadleaf trees
ENL	evergreeen needleleaf trees
U	urban/built up
